# Supplementary material for: Sequence analysis of SARS-CoV-2 genome reveals features important for vaccine design
Source: Sci Rep. 2020 Sep 24;10:15643. doi: 10.1038/s41598-020-72533-2 (PMC7519053; doi:10.1038/s41598-020-72533-2)
Supplement: Supplementary file 1 — Supplementary Information 1. [file 41598_2020_72533_MOESM1_ESM.pdf]

Sequence analysis of SARS-CoV-2 genome reveals features important for vaccine design

Jacob Kames[1], David D. Holcomb[1], Ofer Kimchi[2], Michael DiCuccio[3], Nobuko Hamasaki-Katagiri [1], Tony Wang[4], Anton A. Komar[5], Aikaterini Alexaki[1,*] and Chava Kimchi-Sarfaty[1,*]

[1] Center for Biologics Evaluation and Research, Office of Tissues and Advanced Therapies, Division of Plasma Protein Therapeutics, Food and Drug Administration, Silver Spring, MD, USA

[2] Harvard University School of Engineering and Applied Sciences

[3] National Center of Biotechnology Information, National Institutes of Health, Bethesda, MD, USA

[4] Center for Biologics Evaluation and Research, Office of Vaccines Research and Review, Division of Viral Products, Food and Drug Administration, Silver Spring, MD, USA

[5] Center for Gene Regulation in Health and Disease, Cleveland State University, Cleveland, OH, USA

Corresponding authors:

[*]Chava Kimchi-Sarfaty, Ph.D.; Tel: +1 240 402-8203; Chava.kimchi-sarfaty@fda.hhs.gov; Division of Plasma Protein Therapeutics, Office of Tissue and Advanced Therapies, Center for Biologics Evaluation and Research, Food and Drug Administration, Silver Spring, USA

Correspondence may also be addressed to,

[*]Aikaterini Alexaki, Ph.D.; Tel: +1 240 402 7072; Aikaterini.alexaki@fda.hhs.gov; Division of Plasma Protein Therapeutics, Office of Tissue and Advanced Therapies, Center for Biologics Evaluation and Research, Food and Drug Administration, Silver Spring, USA

Supplemental Figure 1

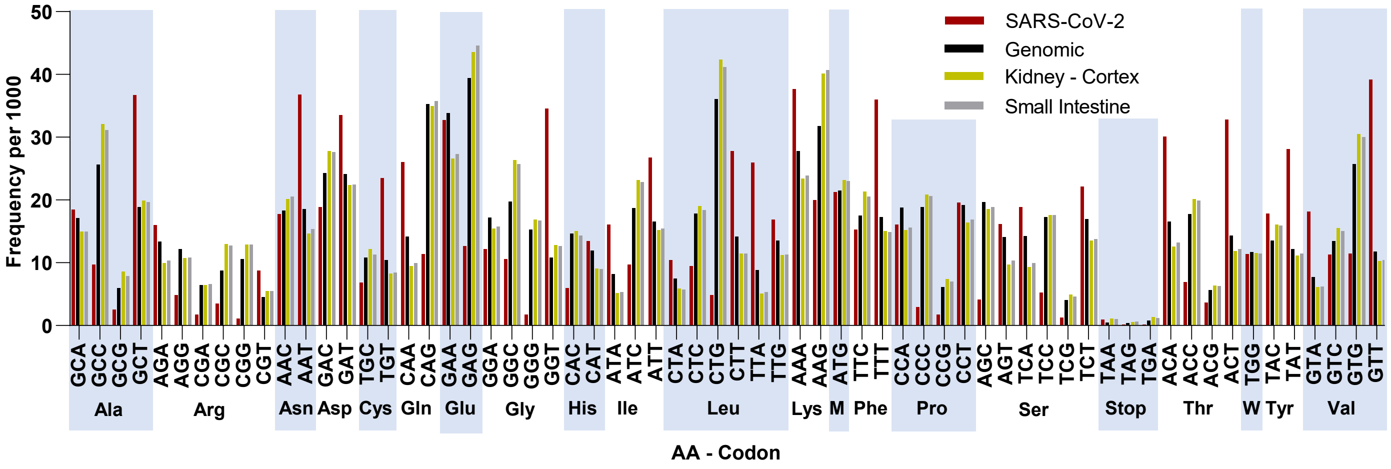

Supplemental Figure 1 – Codon frequencies per 1000 for SARS-CoV-2 (Red), *Homo sapiens* (Genomic, Black), Kidney (Cortex, Yellow) and Small Intestine (Terminal Ileum, grey). Codons are grouped by the amino acid they encode (alternating light blue columns, Met and Trp represented as single letter).

Supplemental Figure 2

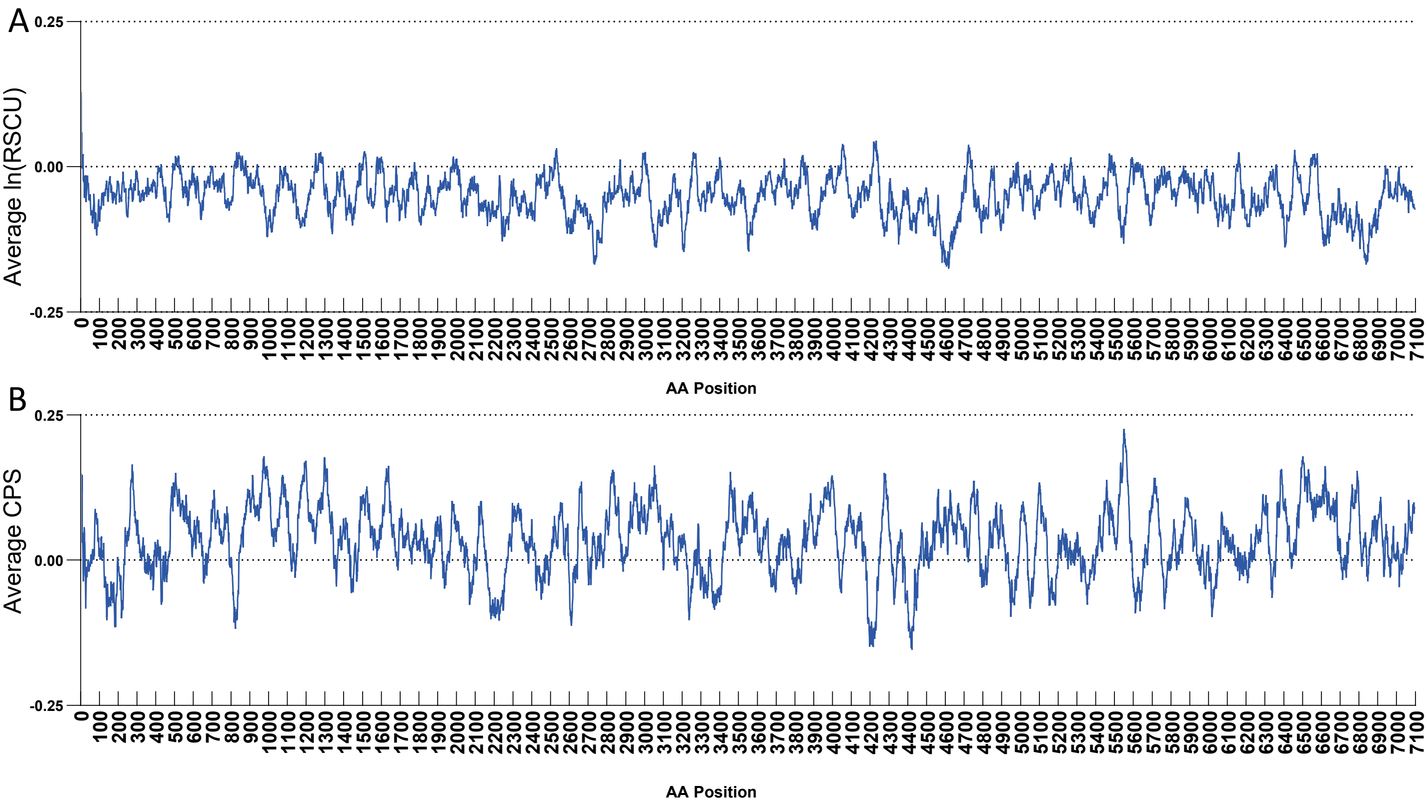

Supplemental Figure 2 – Fifty codon sliding window average of ln(RSCU) (**A**) and codon pair score (CPS) (**B**) of SARS-CoV-2 ORF1ab. RSCU and CPS are calculated based on *Homo sapiens* genomic codon and codon pair usage.
